# Conference Report: SECOND WORKSHOP ON INDUSTRIAL APPLICATIONS OF SCANNED PROBE MICROSCOPY Gaithersburg, MD May 2–3, 1995

**DOI:** 10.6028/jres.101.009

**Published:** 1996

**Authors:** John A. Dagata, Alain C. Diebold, C. K. Ken Shih, Richard J. Colton

**Affiliations:** Precision Engineering Division, National Institute of Standards and Technology, Gaithersburg, MD 20899-0001; SEMATECH, 2703 Montopolis Road, Austin, TX 78714; Department of Physics, University of Texas, RLM 5.208, Austin, TX 78712; Code 6177 Naval Research Laboratory, Washington, DC 20375

## 1. Introduction

The Second Workshop on Industrial Applications of Scanned Probe Microscopy (IASPM) was held at the National Institute of Standards and Technology, on May 2–3 1995 [1]. The meeting, co-sponsored by NIST, SEMATECH, the American Society for Testing and Materials (ASTM) E42.14 Subcommittee, and the Manufacturing Science and Technology Group of the American Vacuum Society (AVS), was attended by approximately one hundred scanned probe microscopy (SPM) users, suppliers, and researchers from industry, government, and academia.

The workshop featured over fifty industry overviews and technical presentations of SPM-based research and development (R&D) activities arranged into three oral focus sessions and poster presentations. A fourth session was devoted to progress on SPM measurement standardization and tool development issues which have occurred since the previous workshop.

### 1.1 Workshop Motivation

SPM-based methods, because of their inherent electro-mechanical simplicity and their capability for making highly localized measurements of surface topography and other properties, represent an attractive opportunity for nonincremental improvements in measurement technology for process development and process control in a variety of advanced manufacturing applications. This has created a very dynamic situation where the deployment of SPMs for a number of critical applications is now underway in US industrial R&D labs. Growth in SPM sales has increased dramatically in the past few years, from only a few units in 1989, to numbers approaching or exceeding a thousand in 1995. A substantial portion of this growth is industrially based, and SPM vendors are shifting their market focus accordingly. As industrial experience with this tool is gained, additional applications, driven by the need for decreasing dimensional tolerances and higher performance of parts and materials, are expected to bring SPM from the R&D laboratory into the production environment [2].

Quantitative measurements represent a foundation upon which commercial acceptance of SPM-based process control and development is grounded. The IASPM workshop aims to foster effective development of standards documents and tool development as a means of achieving widespread industrial acceptance of SPM-based methods. Acceptance of these methods within individual operating units of a company or among firms demands that these tools meet performance standards specific to the application. While it would be preferable that standards development proceed in parallel with the introduction of this new industrial measurement technology, history shows that this is not the case and, in fact, there is a substantial lag time between the initial application of a new technique and the adoption of standard practices associated with its use.

Preparing the applied SPM community for such consensus, the IASPM workshops promote the formation of a network for improved communication among members of the applied SPM community. Passage of new technology from the basic research laboratory to the production facility is difficult today because of the high level of investment required on the part of individual firms to channel basic research results into their R&D pipeline. Current SPM applications are limited to one or a few steps of a complex manufacturing stream, making it unlikely that a single industrial firm will be willing to invest in development efforts thought to be premature. An informed view of the SPM development path, shared by SPM vendors, industry users, and research personnel in university and government laboratories, is critical for widespread industrial acceptance of SPM methods.

The workshops are an effort to identify the roles and responsibilities of each of these member groups of the applied SPM community. The ultimate benefits of widespread industrial acceptance are to be shared by SPM manufacturers and their industrial customer base. It is only as a consequence of bringing about a clear understanding of their roles in the SPM standards and tool development processes, supported by government and university involvement whenever appropriate, that the efforts of the IASPM workshops and standards organizations will be worthwhile.

### 1.2 Workshop Objectives

The objectives of this workshop were:

- To assess progress which had occurred in the past year on SPM standardization and development needs identified in the Summary Report of the first IASPM workshop [3].
- To conduct Focus Sessions limited to Industrial Applications of SPMs in the areas of Magnetic Data Storage Technology, Polymers and Coatings, and Semiconductors.

### 1.3 Purpose of this Report

Part I of this report presents a summary of the major themes which emerged during the technical workshop presentations and discussions. Generic issues transcending individual industrial applications form the basis for an evaluation of overall progress on SPM standardization and instrument development. These are identified and discussed in this report. During the past year, for example, NIST has responded to the needs identified at the first IASPM workshop by carrying out highly regarded standardization efforts on tip deconvolution and step-height calibration. SPM vendors have implemented, or are in the process of implementing, many of the critically needed instrument and software features. Part I concludes with a recommendation for a third workshop. Part II of this Report reprints the workshop Program, Extended Abstracts for all oral and poster presentations, and a Participants List.

## 2. Overview of the Workshop Presentations

### 2.1 Magnetic Recording Technology Session

The workshop program began with a session on Magnetic Data Storage Technology, organized by John Moreland of NIST Boulder. An overview of SPM applications in this industry was given by Allan Schultz of Seagate Technologies. As bit densities exceed 1 Gbit-in2, new techniques are needed for imaging the fields very near media surfaces and the airbearing surfaces (ABS). Bit transitions separated by less than 500 nm and head flying heights of less than 20 nm will be typical of disk drives in the next 5 years. The industry needs for nanometer-scale tolerances during component fabrication and testing critical components of a disk drive include pole tip recession of the read/write head, the domain structure of the magneto-resistive (MR) sensor, the relationship of grain size and surface roughness on the magnetic bit patterns, especially the fringe fields at the edges, and the wear properties of lubricants coating the disk surface which mediate the contact between the ABS and disk surfaces.

SPM topographic measurements of pole tip recession during quality assurance and acceptance testing were discussed in a talk by Jim Potter of Maxtor, the potential of magnetic force microscopy coupled with topographic imaging was discussed by Dan Dahlberg and Yansheng Luo of the University of Minnesota and Ken Babcock of Digital Instruments, and use of the SPM for determining the normal and lateral interaction forces which influence tribology in disk drives was discussed in a talk given by Mathew Mate of IBM Almaden.

The following themes emerged during the session: First, there was significant interest noted in SPMs for performing not only topographic imaging, but other measurements simultaneously. In the topographic mode, SPM is being used for critical dimensioning of the ABS, and allows for direct observation of media roughness at wavelengths as low as a few nanometers. Specific manufacturing issues are that polishing of hard disk substrates can be monitored with high spatial resolution, zone-texturing of disks can be optimized to minimize head wear, and grain morphology and size can be estimated.

In addition to topographic information, Magnetic Force Microscopy (MFM) is used to image the stray fields near bits recorded in media and near the ABS. It can also be used to image the domains in thin-film permalloy MR sensors, permanent magnetic films used to bias the MR sensors, and the soft magnetic films used to shield the MR sensors. Currently, measurements with the MFM are limited to relative comparisons typically between different samples with the same tip. Examples include, fringing fields above bits written in different media with the same head to determine relative media recording parameters, or fringing fields near the ABS of heads energized at different current levels to determine head saturation levels.

Lateral Force Microscopy (LFM) is being used to study the properties and performance of the lubricating layer deposited on media surfaces to minimize damage during head-media contact. Lubrication coverage uniformity can be imaged directly with LFM. Friction creates a lateral force acting on the SPM cantilever and media lubricant which can be resolved with nanometer spatial resolution. Some knowledge of the chemical nature of the tip surface is required before LFM images can be related to molecular frictional processes; however, a qualitative picture of contact processes between an SPM tip and surface lubricants can be developed. Hysteresis of the force associated with dipping the SPM cantilever into the lubricant, pushing lubricant out of the way, and subsequently pulling free of the lubricant, can be measured in this way with a sensitivity of better than 10^−10^ N.

Second, there is a divergence in the way industry uses SPMs for production quality control and in process control and development. Research applications include fundamental studies of new kinds of storage media and thin-film recording heads. Specifically, SPM has been used to correlate magnetic media noise and grain morphology, as well as domain structure, in as-deposited films and films patterned into simple devices. Product development applications include studying the recording process in various head-media combinations and testing of component wear. Quality assurance applications include spot-testing of individual components from batches manufactured for assembly and testing of components shipped for assembly from other companies.

Third, industry needs relative as well as absolute calibration for MFM calibration standards. In addition to the general needs of the community for topographic standards and tip de-convolution, an artifact standard for MFM measurements would be useful for testing instrument sensitivity and resolution. It would be helpful if the standard was flat, thereby avoiding imaging artifacts such as topographic influences on the MFM image. The standard would require rapid field gradient changes at a period of 1mm, or smaller. As in the case of comparing SPM to other profiling and roughness measurement techniques, the advantages and disadvantages of MFM relative to other magnetic imaging techniques needs to be clarified.

### 2.2 Polymers and Coatings Session

The second session of the workshop was entitled Polymers and Coatings, organized by Andy Gilicinski of Air Products and Chemicals. An overview of SPM applications in the polymer and coatings industry was given by Greg Blackman of DuPont. The issue has gone from is it possible to image the surfaces of soft materials’ such as polymers to the more significant one of using SPM-based methods to better understand how the micromechanical properties of (complex) polymers systems affect the performance of films and coatings with respect to their intended purpose.

The main focus of current efforts in the applications concerned new methods for achieving high resolution chemical contrast by Don Chernoff of Advanced Surface Microscopy, obtaining and understanding the relationship between SPM image and polymer structure presented by Gerry Zajac of Amoco, using the SPM to understand the control of particle-film morphology during growth by Cynthia Goh of the University of Toronto, quantitative measurements of adhesion by Howard Mizes of Xerox, indentation by Greg Meyers of Dow Chemical, and the use of force modulation measurements for distinguishing between different phases in polymer blends by Andy Gilicinski.

There were several common themes which emerged during the session: First, it is clear that topographic imaging, coupled with other modes which can provide chemical and mechanical information about the film properties, is considered to be a critical aspect for process development. The widespread availability now of noncontact modes of operation for AFM which provide high-resolution imaging without damage to the sample surface are combined in commercial instruments with friction, lateral force, and force-modulation modes. Although it may be impossible to obtain data from all modes simultaneously, sequential scanning over a single region of the sample surface following a change of hardware or software is now generally possible with the current generation of SPMs.

The significance of multiple mode sampling was evident from several of the session presentations. Key criteria for evaluating the performance of coatings and polymer additives included: adhesion, wettability, friction, wear, stiffness, scratch damage, and appearance. Interpretation of these criteria requires direct and systematic correlation of molecular weight, film forming properties, and solvent content during process control and optimization.

Second, quantifying this information, particularly the correlation of micro-mechanical properties is becoming a serious concern in industry. Some of the current effort is devoted to model systems which are amenable to quantitative analysis. Investigations of real commercial systems, as in the use of SPM imaging to reveal blends of co-polymer coating systems, suggest the range of SPM applications for predicting wear, adhesion, and film thickness properties.

Third, standards for characterizing tip shape and determining its effect on image resolution remain an important issue, not only for topographic data related to polymer systems, but for the realization of quantitative lateral-force imaging in the near future.

### 2.3 Semiconductor Session

The first IASPM workshop focused largely on the needs of the semiconductor industry for topographical inspection. These needs involve microroughness for wafer acceptance testing and critical dimension (CD) measurement in lithography. The present workshop emphasized using SPMs for in-line and off-line defect review and CD control. It is significant that only during the past year has it become possible to hear about actual user experience. Neal Sullivan of Digital Equipment Corporation discussed acceptance and qualification of an in-line tool for particle review, defect inspection, 1-D and 2-D CD metrology, grain-size evaluation of metallization layers, and planarization. Henry Luftman of AT&T Bell Labs discussed the use of SPM for off-line analysis of materials problems and planarization.

The major themes which emerged from this session were: First, SPM systems are now being offered commercially which may be integrated with in-line optical inspection stations, data exchange of *x* and *y* coordinates, and particle size. Automation of system calibration and pattern recognition sequences, as well as topographical measurement, analysis, and data transfer, are available or close to being offered on the latest high-end systems. Yale Strausser of Digital Instruments (DI) described strategies which DI considers promising in this regard.

Second, the development of SPM methods for electrical characterization, topic of the first workshop, was revisited in terms of the scanning Kelvin probe microscopy (SKPM), as reported by Martin O’Boyle of IBM. The SKPM methods provide information on the presence of contaminants which alter the electrical properties of metallization layers, or change the interfacial properties at ohmic, Schottky, or insulator regions. This probe makes it possible to correlate device failure or abnormal device performance with a localized difference in potential, thereby narrowing the problem down to a specific process step.

Third, Near-field Scanning Optical Microscopy (NSOM) reached the status of a commercial instrument during the past year. This provides an alternative to the mechanical capability of SPM techniques, relying rather on optical techniques. Herschel Marchman and Walter Duncan of Texas Instruments described the needs and directions which the semiconductor industry is investigating for using NSOM as an *in-situ* lithometrology tool. In particular, the NSOM technique may have a significant role to play in process development by providing critical dimension information on exposure parameters in the latent resist image, process parameters during bake and etch steps, as well as on dimensional control of the final patterned resist. The NSOM is also promising for defect identification of sub 0.1 μm particles by using it in a spectroscopic mode, i.e., photoluminescence, fluorescence, or Raman. Grover Wetsel of the University of Texas-Dallas and Paul West of Topometrix discussed some aspects of instrument evolution in this rapidly developing field.

Despite the progress on in-line instrumentation, SPM techniques must prove their value to industry first in off-line process control applications. The instrumentation must be flexible, yet the routine tasks such as system calibration and tip exchange must become increasingly more rapid, if not automated, allowing R&D personnel to concentrate on developing the appropriate methodology rapidly and systematically. Tip calibration and standards for roughness and step height clearly represent a fundamental step towards this end. Again, there remains a entire spectrum of opportunities between in-line and off-line applications.

### 2.4 Progress on SPM Standardization and Development Needs

A fourth session organized by Jason Schneir of NIST and Joe Griffith of AT&T Bell Labs, provided an opportunity for the Workshop participants to assess the responsiveness of the standards community and SPM vendors towards meeting the needs expressed at the first IASPM workshop.

The major themes discussed at this session were: First, probe-tip deconvolution algorithms and their integration into SPM software is being actively pursued by researchers and vendors. At this time, detailed model calculations need to be done to give validity to more approximate methods which would be faster and more amenable to commercial applications. John Villarrubia of NIST discussed progress on tip modeling. The SPM tip is fairly well recognized as one of the greatest potential sources of variability in measurements.

This is certainly true in the simplest case of purely geometric considerations, but it was noted that variations in the orientation and quality of the magnetic thin-film coating on MFM tips, for example, might be responsible for large variations in signal intensity observed experimentally. Since microfabrication techniques seem to produce fairly uniform tip geometries on the wafer, either better process control of the deposition process is necessary or an identification of the cause based on careful modeling of the interaction of the MFM tip with a “known” standard must be pursued.

Second, another probe-related issue which arose during this workshop was the use of spherically shaped objects for micromechanical experiments since for this geometry contact experiments measuring indentation or adhesion are more amenable to calculation. Given the recognized importance of mechanical information for the polymer and coatings industry, these probes must be considered part of the ongoing interests of this workshop. Likewise, NSOM probes were discussed. The exciting, although early, progress on using photoluminescence, fluorescence, and Raman techniques for spectroscopic evaluation of sub 0.1 μm defects and mapping strain in device structures will also require probes with sufficient light throughput to achieve reasonable signal-to-noise ratios using these techniques. In general, the developments suggest that for many industrial applications sharper tips may not be so important as ones that can be used quantitatively or that yield more reproducible characteristics on a tip-to-tip basis.

Third, the development and widespread availability of suitable standardized roughness and step-height calibration samples is needed. Industry requirements were addressed by Mark Lagerquist of IBM. Progress on step-height standards was reported by Jason Schneir of NIST. It is clear that future workshops must provide the participants with a formal program which outlines the various aspects of standards development. NIST, in particular its Precision Engineering Division (PED), has expressed its intent to support SPM standards activities. In addition, there is much to be learned from existing profilometer and scattering step height and roughness standards. However, the point was made several times during the workshop that often an “in-house” reference which is deemed “good” is used for relative comparisons of SPM signal calibration. For many applications this will be good enough.

Fourth, the timely publication of standard methods and practices for obtaining and reporting experimental data is needed. Dennis Swyt, Chief of PED, gave an overview of how PED performs its role in providing for SPM calibration and standardization needs. Alain Diebold of SEMATECH, chair of the ASTM E42.14 subcommittee reported on the status of activities for which that body is responsible.

## 3. Discussion

The first IASPM workshop identified several areas of SPM performance where advances were needed: the universal availability of linearized piezo-scanners over the entire SPM product line, automated sampling for statistical process control, improved probe characteristics including tip-shape and wear properties, and a more complete understanding of the underlying physical and chemical interactions which affect sensitivity and image contrast.

The goal of the first Workshop was aimed at identifying developments which would impact on the quantitative aspects of topographic SPM-based measurement. Recall that the facilitated discussions following each of the three focus sessions were concerned with Microroughness, Critical Dimension Metrology, and Electrical Characterization. The discussions focused on specific targets given in the 1992 Semiconductor Industry Association (SIA) Roadmap, and some information from the draft 1994 SIA Roadmap, in order to define the standard of performance required by the semiconductor industry for SPM tools over the next decade [4].

The second Workshop examined a broader range of applications than the first. Friction, lateral force, optical, magnetic force, nanomechanical, and nanoindentation measurements, often recorded simultaneously in conjunction with a topographic image, is an extremely valuable, perhaps essential, aspect of SPM strategies for process development. Enhanced functionality, and the information made possible by it, is a significant value-added feature of SPMs. These features are most significant for process development applications.

Experience with dedicated tools for high-throughput inspection for semiconductor industry applications is accumulating. Critical dimension and defect review represent the primary measurements which these tools will perform. Topographic AFM data and its quantification therefore remain the most immediate and generic SPM needs.

The most general use for SPMs will continue to be for gathering topographic information. Quantification of surface microroughness and texture as well as dimensional measurements such as step height and pitch, remains the key standardization issue for the SPM community. There has been sustained progress in this area and in SPM tip characterization as well. This progress has emerged from considerable activity at NIST and elsewhere on tip and sample calibration and understanding tip shape and wear, for example.

A common theme which several of the workshop speakers emphasized at all three focus sessions was the importance of simultaneous collection of topographic SPM data together with additional surface properties. Electrical potential, magnetic, chemical, friction, lateral force, index of refraction, nanohardness are examples of such properties. A map of surface, or near-surface, variations in physical structure or composition may be the primary goal of a measurement, as in magnetic-force measurements. It may provide a means of detecting unexpected problems in materials processing of polymer blends or sources of contamination during quality control testing. The interplay of such complementary information proves advantageous, if not necessary, under these circumstances.

In the first case, a topographic map may be needed to subtract out variations in the MFM image due to the convolution of surface topographic structure intermixed with the magnetic-signal map. Alternatively, chemical-phase or contaminant identification by friction, in the case of lateral force microscopy, or electrical potential variations in the case of SKPM, may be performed during failure analysis making this an extremely valuable feature for process development or defect inspection. The increasing flexibility of commercially available general-purpose SPMs to operate in a variety of simultaneous modes is unique among surface analytical instruments. As all speakers mentioned, however, these strategies, still largely under development, require additional correlation with other techniques before they can be used with confidence. Future workshops may consider the need and development path for other quantitative standards, e.g., magnetic or nano-hardness standards.

The lab to fab transition is now underway. From the time of the first IASPM workshop to the time of the second workshop, several semiconductor companies have taken delivery of in-fab SPM tools. Additional SPM vendors are now also offering highly automated tools for dedicated wafer inspection. These systems offer features such as coordinate transfer between current generation, on-line optical inspection stations, and the SPM tool, automated operation, and pattern recognition software.

R&D on model systems is proceeding in industrial labs and at universities. Specific examples discussed at the workshop were MFM on magneto-bacteria and adhesion studies in the polymer area. Model systems are critical for the development of analytical data treatment and for quantification. This is an area where communication between industry researchers and university/government researchers can result in fruitful collaborative efforts.

A grasp of these technological factors alone is not sufficient for evaluating the progress and acceptance of SPM-based methods by industry. R&D-driven innovation has become more difficult because of the high level of investment required on the part of individual companies to perform a meaningful level of basic research within their own laboratories and a decreasing capacity for purely technological advantages to offer significant returns on investment. As the focus of most current corporate R&D has moved towards short-term goals, it has become more difficult for industry to perform the kind of R&D required to ensure that SPM-based tools meet the performance standards demanded by advanced manufacturing over the next decade.

Furthermore, there is a sense that traditional methods for creating standards documents may be less effective in the present environment of industry restructuring and government downsizing. Progress depends on the sustained volunteer activity of many individuals who take on the responsibility of identifying the consensus areas and patiently writing and sheparding the documents through the organizations. While this approach has worked in the past, industrial researchers have little time available to devote to such altruistic efforts. Government personnel also have difficulty committing to this process. Without a substantial push from the applied SPM community as a whole, a consensus on standards and standard practices will materialize slowly, despite the acknowledged interest in and importance of these issues. With few people able to assume a leadership position in standards activities and with the pressure on industry performance, there may be few opportunities to achieve a consensus on standards documentation for SPM applications.

Finally, SPM development is occurring at a very early stage in the life of these instruments. It is well known that when technological needs drive developments in a new field, the scientific understanding underlying the technique generally lags behind the use of the technique as long as its use provides a meaningful advantage in manufacturing. At the same time there are several manufacturers of SPMs which have entered this market beginning for the most part as suppliers to a research-oriented clientele. As these suppliers introduce products into industrial settings, standards of performance begin to become crucial. The United States, as the home of the major suppliers of industrially oriented SPMs, is in a position to benefit in a significant way from the early and successful introduction of SPM-based technology into advanced manufacturing.

## 4. Recommendation for a Third IASPM Workshop

The foregoing section discussed some of the implications of current standards and tool development efforts which emerged during the second IASPM workshop. To summarize:

- The simultaneous collection of SPM topographic data with other surface properties, e.g., electrical potential, magnetic, optical, chemical, mechanical, friction, etc., provides a strong value-added element for industrial applications, particularly in process development and off-line defect review.
- SPM topography issues, specifically tip characterization and dimensional calibration of roughness and step height, remain high-priority standardization needs. Although the SPM requirements for process development tools differ from those required for high-throughput in-line metrology, performance standards which quantify topographic measurements are common to both applications.
- Without a concerted effort on the part of the applied SPM community, documents which establish standards and standard practices for SPM use will be developed in a very slow manner despite the importance of these documents for the advancement of SPM methods in industry.

Based on these observations, we recommend that a third workshop be held at NIST Gaithersburg in 1996. The workshop format will focus on two well-defined objectives: First, a substantial part of the meeting time will be devoted to an in-depth tutorial on standardization activities. Some of the topics under consideration include: Understanding the relationship between standards documents, practices, artifacts, and calibration; accounting for the unique challenges involved in defining nanometer-scale standards and procedures; explanation of how standards documents are written and a consensus established; overview of standards organizations actively involved in SPM-related projects; discussion of the overlap which future SPM standards may have with existing optical- and stylus-based measurements of surface roughness and step-height measurements; and, the relevance of ISO 9000 conformance. Formal discussion periods following the tutorial will allow participants to lay the groundwork for formal SPM standard document activity.

A second objective of this workshop will be to again present progress reports in the applications areas which have been covered in previous focus sessions, semiconductors, data storage, and polymers, as well as in other areas where there is a significant industrial interest. The workshop format will continue to serve as a filter for recent advances and development issues in both in-line metrology and process development applications. Efforts are underway to form an industry user–SPM vendor advisory committee to help identify emerging and novel SPM-based strategies and methods in industrial applications for presentation during the workshop.
